# Dynamic Equilibria between DNA-Stabilized Silver Nanoclusters and Silver-Carrying DNA Strands

**DOI:** 10.1021/acs.jpclett.5c02324

**Published:** 2025-10-09

**Authors:** Cecilia Cerretani, Donato Ranieri, Giacomo Romolini, Christian Brinch Mollerup, Letizia Liccardo, Elena-Alexandra Niţă, Loredana Latterini, Tom Vosch

**Affiliations:** † Department of Chemistry, 4321 University of Copenhagen, Universitetsparken 5, 2100 Copenhagen, Denmark; ‡ Department of Chemistry, Biology and Biotechnology, Perugia University, Via Elce di Sotto, 8, 06123 Perugia, Italy; § Department of Forensic Medicine, University of Copenhagen, Frederik V’s Vej 11, 2100 Copenhagen, Denmark

## Abstract

DNA-stabilized silver nanoclusters (DNA-AgNCs) are versatile emitters whose photophysical properties are defined by the DNA template. In this study, we explore the unusual temperature- and concentration-dependent behavior of two NIR-emissive DNA-AgNCs: one stabilized by two 16-base DNA oligomers (16mer), and another embedded within a 3-base shortened version of these strands (13mer). Using a combination of optical spectroscopy and mass spectrometry, we show that Ag^+^-carrying DNA strands can reversibly attach to DNA-AgNCs and fine-tune their photophysical properties. For 13mer-AgNC, we demonstrate the presence of three different species, (13mer)_2_-[Ag_20_]^10+^, (13mer)_3_-[Ag_27_]^17+^, and (13mer)_4_-[Ag_34_]^24+^, depending on concentration and temperature. For the AgNC stabilized by two 16-base DNA strands, (16mer)_2_-[Ag_20_]^10+^, emission wavelength and intensity vary with temperature, although no significant concentration-dependent spectral shifts are observed. While mass-spectrometry revealed the existence of (16mer)_3_-[Ag_27_]^17+^, time-resolved anisotropy uncovered the formation of aggregates of (16mer)_2_-[Ag_20_]^10+^. These findings demonstrate the presence of dynamic equilibria between Ag^+^-carrying DNA strands and DNA-AgNCs.

## Graphical abstract

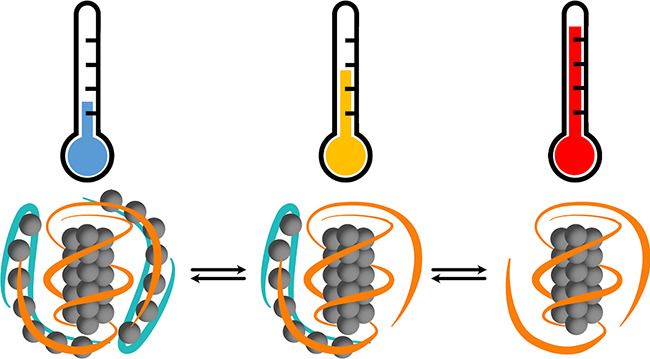

DNA-stabilized silver nanoclusters (DNA-AgNCs) are emitters composed of 10–30 Ag atoms and cations stabilized by one or more single-stranded DNA oligomers.^1–4^ The DNA sequence serves as a template, defining the size, shape and charge of AgNC and thus determining its photophysical properties. DNA-AgNCs are often rodlike in shape and even numbers of valence electrons from 4 to 12 have been reported for green to near-infrared (NIR) emissive clusters.^5,6^ In this study, we report the unusual behavior of two NIR emissive DNA-AgNCs with a compositionally similar core of 10 valence electrons that only differ in the length of the stabilizing DNA strand. The investigated DNA-AgNCs were synthesized using a previously reported 16-base sequence, 5′-CCCA­CCCA­CCCT­CCCA-3′ (16mer), known to produce an 820 nm emissive (16mer)_2_-[Ag_20_]^10+^,^7–12^ and a shortened 13-base sequence, 5′-CCCAC­CCAC­CCTC-3′ (13mer), introduced here, which stabilizes a similar but more intriguing (13mer)_2_-[Ag_20_]^10+^ emitter. Details on the synthesis and HPLC purification can be found in the Supporting Information and Figures S1 and S2.

As shown in Figure 1, both DNA-AgNCs exhibit absorption and emission spectra in a similar wavelength range. At 25 °C, the absorption maximum for 16mer-AgNCs is at 758 nm and the emission is centered at 829 nm, while for 13mer-AgNCs, an absorption maximum at 740 nm and emission centered at 800 nm were found. However, upon varying the temperature from 5 to 40 °C, the absorption maximum of 16mer-AgNCs stays constant, while for 13mer-AgNCs it blue-shifts 25 nm (460 cm^–1^) from 10 to 40 °C. Surprisingly, for both 16mer-AgNCs and 13mer-AgNCs, the emission maxima blue-shift and increase in intensity when increasing the temperature. This behavior is most pronounced for 13mer-AgNCs, with a blue-shift of 22 nm (346 cm^–1^), when going from 10 to 40 °C (see Figure 1B), whereas for 16mer-AgNCs the blue-shift amounts to 131 cm^–1^ (from 5 to 40 °C). Absorption and emission maxima at different temperatures are summarized in Table 1. Additionally, we verified that the changes in the position of the absorption and emission spectra for both the 13mer-AgNCs and 16mer-AgNCs were reversible and not caused by the disassembly of the samples upon heating (Figures S7 and S8).

**Figure 1:**
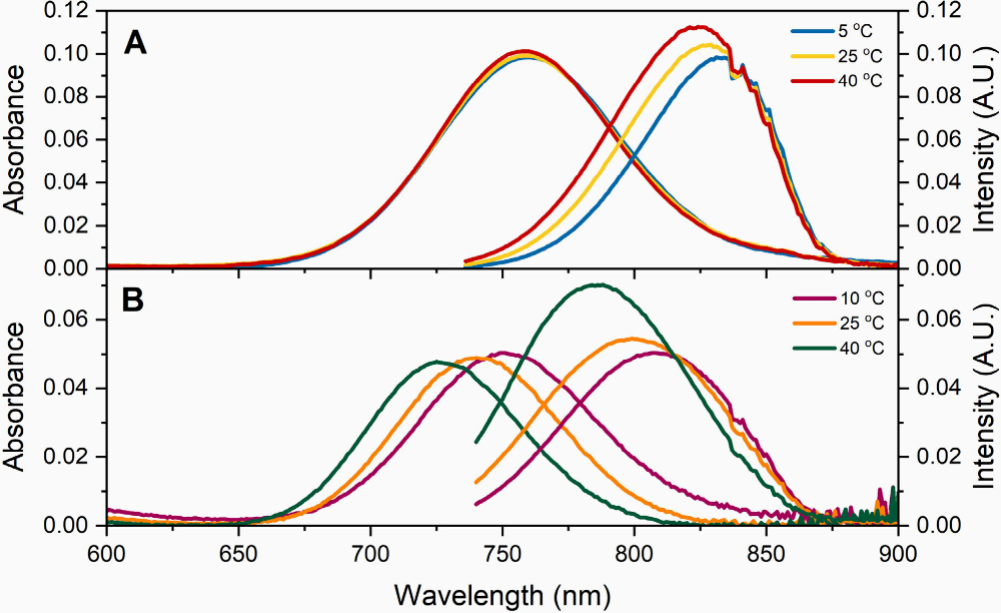
(A) Absorption and emission spectra of 16mer-AgNCs in 50 mM ammonium acetate (NH_4_OAc) at 5, 25, and 40 °C. The concentration of 16mer-AgNCs is 5.6 × 10^–7^ M. (B) Absorption and emission spectra of 13mer-AgNCs in the same medium at 10, 25, and 40 °C. The concentration of 13mer-AgNCs is 2.9 × 10^–7^ M. Emission spectra were recorded exciting at 726 nm. Extended versions of the absorption spectra down to 260 nm can be found in Figure S6.

**Table 1:** Steady-State Absorption (λ_abs_) and Emission Maxima (λ_em_) of 13mer-AgNCs with a Concentration of 2.9 × 10^–7^ M and 16mer-AgNCs with a Concentration of 5.6 × 10^–7^ M in 50 mM NH_4_OAc at Different Temperatures

	13mer-AgNC		16mer-AgNC	
temperature (°C)	λ_abs_ (nm)	λ_em_ (nm)	λ_abs_ (nm)	λ_em_ (nm)
5			760	834
10	750	808		
25	740	800	758	829
40	725	786	758	825

Although unusual, such an emission response is observed in cases where thermally activated delayed fluorescence (TADF) occurs;^13^ however, our data can rule out this option. Besides a blue-shift of the emission spectrum with increasing temperature, TADF should also display an excited-state lifetime component corresponding to the decay time of the long-lived state from where the TADF originates. However, when measuring fluorescence decays, no background signal that can be attributed to a long-lived state (Figures S4A and S4C) was observed, nor was any microsecond-lived luminescence detected using the burst mode approach (Figure S4B). As such, the unusual temperature-dependent intensity increase and emission blue-shift must have another mechanistic origin.

The first clues in unraveling the origin of this unusual behavior came from measuring absorption and emission spectra of 13mer-AgNCs at different concentrations. In 50 mM ammonium acetate (NH_4_OAc), increasing the 13mer-AgNC concentration resulted in a red-shift of both the absorption and emission maxima (Figure 2B), accompanied by a significant, progressive decrease in fluorescence quantum yield (Table 2), while fluorescence lifetime changed only within the experimental error (Table 3).

**Figure 2:**
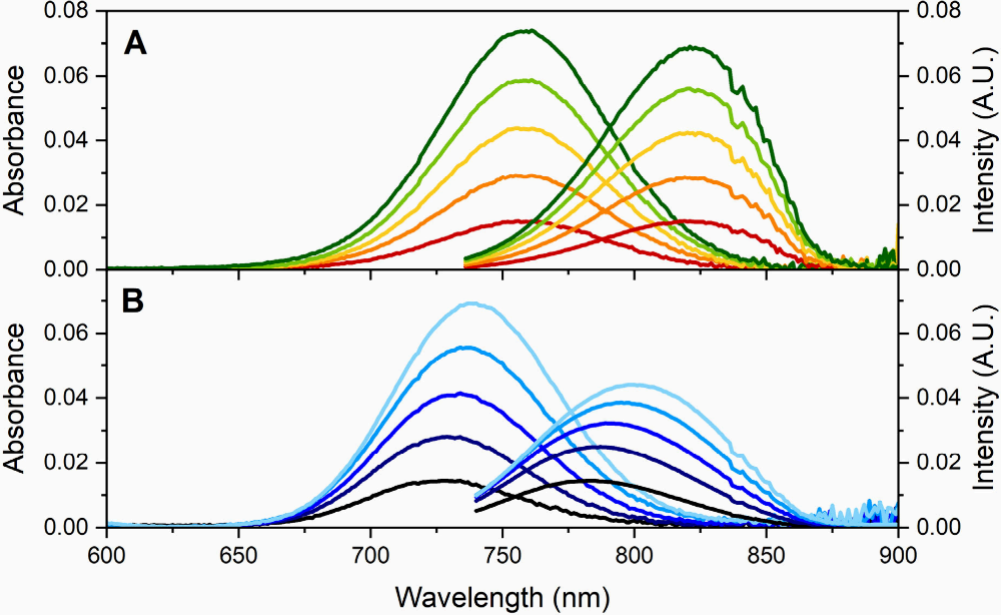
Concentration-dependent absorption and emission spectra of (A) 16mer-AgNCs and (B) 13mer-AgNCs in 50 mM ammonium acetate at 25 °C. Emission spectra were recorded exciting at 726 nm.

**Table 2:** Steady-State and Time-Resolved Data of 13mer-AgNCs at 25 °C in 50 mM NH_4_OAc at Different Concentrations

concentration (M)	Φ
8.4 × 10^–8^	0.65
1.7 × 10^–7^	0.61
2.4 × 10^–7^	0.57
3.2 × 10^–7^	0.54
4.0 × 10^–7^	0.52

**Table 3:** Concentration-Dependent Spectroscopic Properties of 13mer-AgNCs in 50 mM NH_4_OAc at 25 °C^a^

concentration (M)	λ_abs_ (nm)	λ_em_ (nm)	⟨τ⟩ (ns)	θ (ns)	*V* (nm^3^)
8.4 × 10^–8^	730	787	2.13	3.15	14.6
2.2 × 10^–7^	739	800	2.06	4.87	22.5
4.1 × 10^–7^	745	808	2.02	6.76	31.3
8.4 × 10^–8^ (II)	730	788	2.14	3.48	16.1

a Absorption (λ_abs_) and emission maxima (λ_em_) together with intensity-weighted average decay time (⟨τ⟩) measured at 790 nm, exciting at 726 nm. θ indicates the rotational correlation time, while *V* stands for the hydrodynamic volume. (II) represents the measurements of the 4.1 × 10^−7^ M sample diluted to 8.4 × 10^−8^ M to demonstrate reversibility of the concentration effect (see also Figure S5). Details on the time-resolved measurements are reported in the Supporting Information.

Moreover, while the limiting anisotropy value, *r*_0_, of 13mer-AgNC was found to approach 0.4 (Figure S3B), time-resolved anisotropy measurements uncovered a substantial increase in the hydrodynamic volume, from 14.6 to 31.3 nm^3^, at 25 °C, as the concentration was increased from 8.4 × 10^–8^ M to 4.0 × 10^–7^ M (Table 3, Figure 3A). Similar to the concentration-dependent behavior, lowering the temperature of a diluted 13mer-AgNC solution (1.1 × 10^–7^ M) led to a rise in the hydrodynamic volume from 14.8 nm^3^ at 40 °C to 19.8 nm^3^ at 10 °C (Table 4, Figure 3A). These observations led to an early hypothesis of reversible binding processes, hence we employed electrospray ionization-mass spectrometry (ESI-MS) to help elucidate the origin of this unusual behavior.

**Figure 3:**
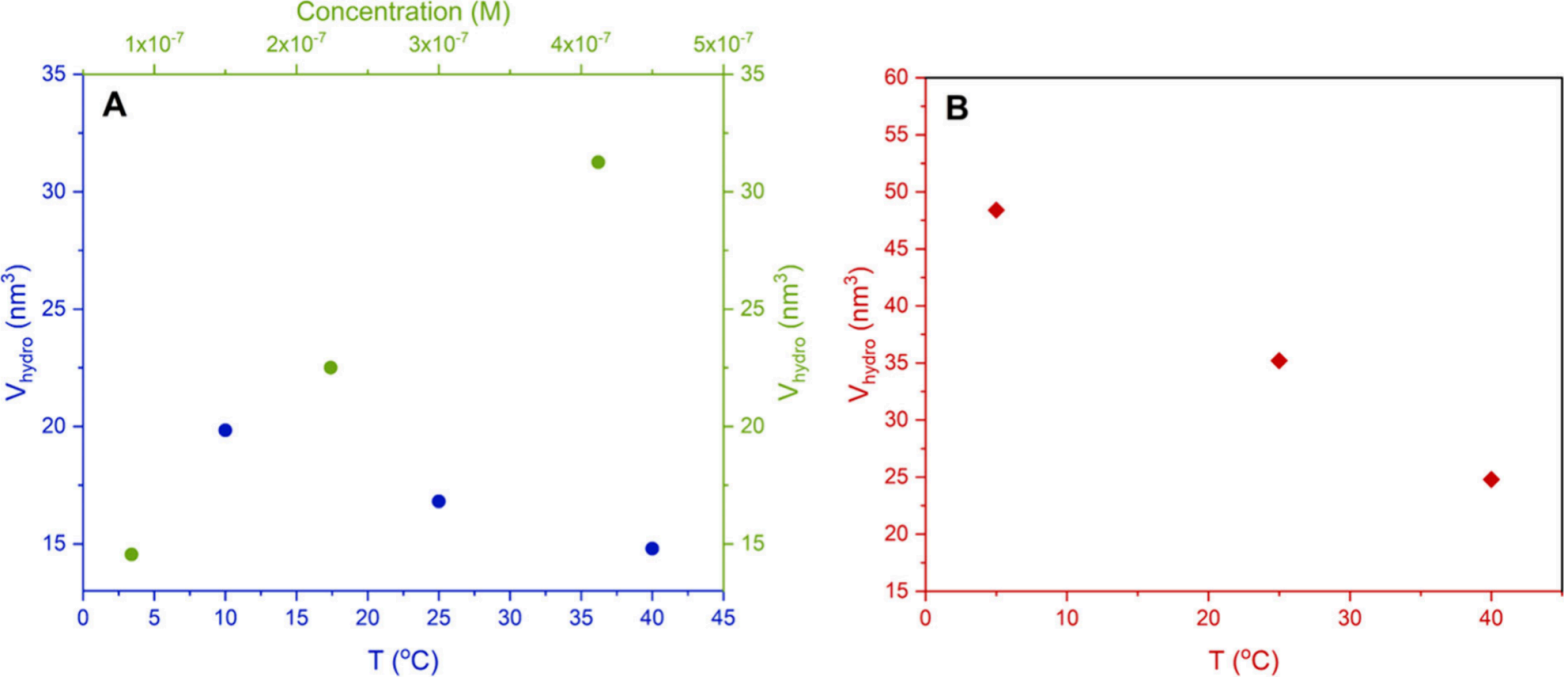
(A) Hydrodynamic volume as a function of concentration (green) at 25 °C and as a function of temperature (blue) for 13mer-AgNC in 50 mM NH_4_OAc. (B) Hydrodynamic volume as a function of temperature (red) for 16mer-AgNC in 50 mM NH_4_OAc (for the cases where the limiting anisotropy reaches 0.4, see also Figure S14). The hydrodynamic volumes calculated at each temperature are 24.8 nm^3^ at 40 °C, 35.2 nm^3^ at 25 °C and 48.4 nm^3^ at 5 °C.

**Table 4:** Steady-State and Time-Resolved Data of 13mer-AgNCs with a Concentration of 1.1 × 10^–7^ M in 50 mM NH_4_OAc at Different Temperatures^a^

temperature (°C)	λ_abs_ (nm)	λ_em_ (nm)	⟨τ⟩ (ns)	θ (ns)	*V* (nm^3^)
10	743	810	2.03	6.64	19.8
25	728	792	2.12	3.64	16.8
40	723	782	2.12	2.24	14.8
25 (II)	729	792			
10 (II)	744	810			

a Absorption (λ_abs_) and emission maxima (λ_em_) together with intensity-weighted average decay time (⟨τ⟩) measured at 790 nm, exciting at 726 nm. θ indicates the rotational correlation time, while *V* stands for the hydrodynamic volume. (II) represents the second measurements done at the specified temperature to demonstrate reversibility of the temperature effect (see also Figure S7). Details on the time-resolved measurements are reported in the Supporting Information.

The mass spectra, shown in Figure 4, as well as Figures S9 and S10, revealed the existence of three distinct species: (13mer)_2_-[Ag_20_]^10+^, (13mer)_3_-[Ag_27_]^17+^ and (13mer)_4_-[Ag_34_]^24+^. We speculate that the actual core compound is (13mer)_2_-[Ag_20_]^10+^ and that, depending on temperature or concentration, one or two DNA strands with 7 silver cations (13mer-[Ag_7_]^7+^) can bind to it. This will most likely involve Ag^+^-mediated base pair interactions,^14–16^ hydrogen bonding and π–π stacking interactions, forming a second shell of DNA on the outside of the (13mer)_2_-[Ag_20_]^10+^ unit (Figure 4D). The aforementioned changes in the absorption and emission spectra are therefore attributed to conformational changes in the surrounding of the emitting core compound, induced upon binding and unbinding of 13mer-[Ag_7_]^7+^ segments to (13mer)_2_-[Ag_20_]^10+^. This can also explain the observed static quenching as the fluorescence quantum yield dropped, whereas the decay time remained constant (Tables 2 and 3). At the same time, the limited emission shift within the same spectral range supports the idea that the Ag^+^ cations in these 13mer-[Ag_7_]^7+^ segments do not merge with the [Ag_20_]^10+^ core, but merely affect the DNA coordination around the [Ag_20_]^10+^ core.

**Figure 4:**
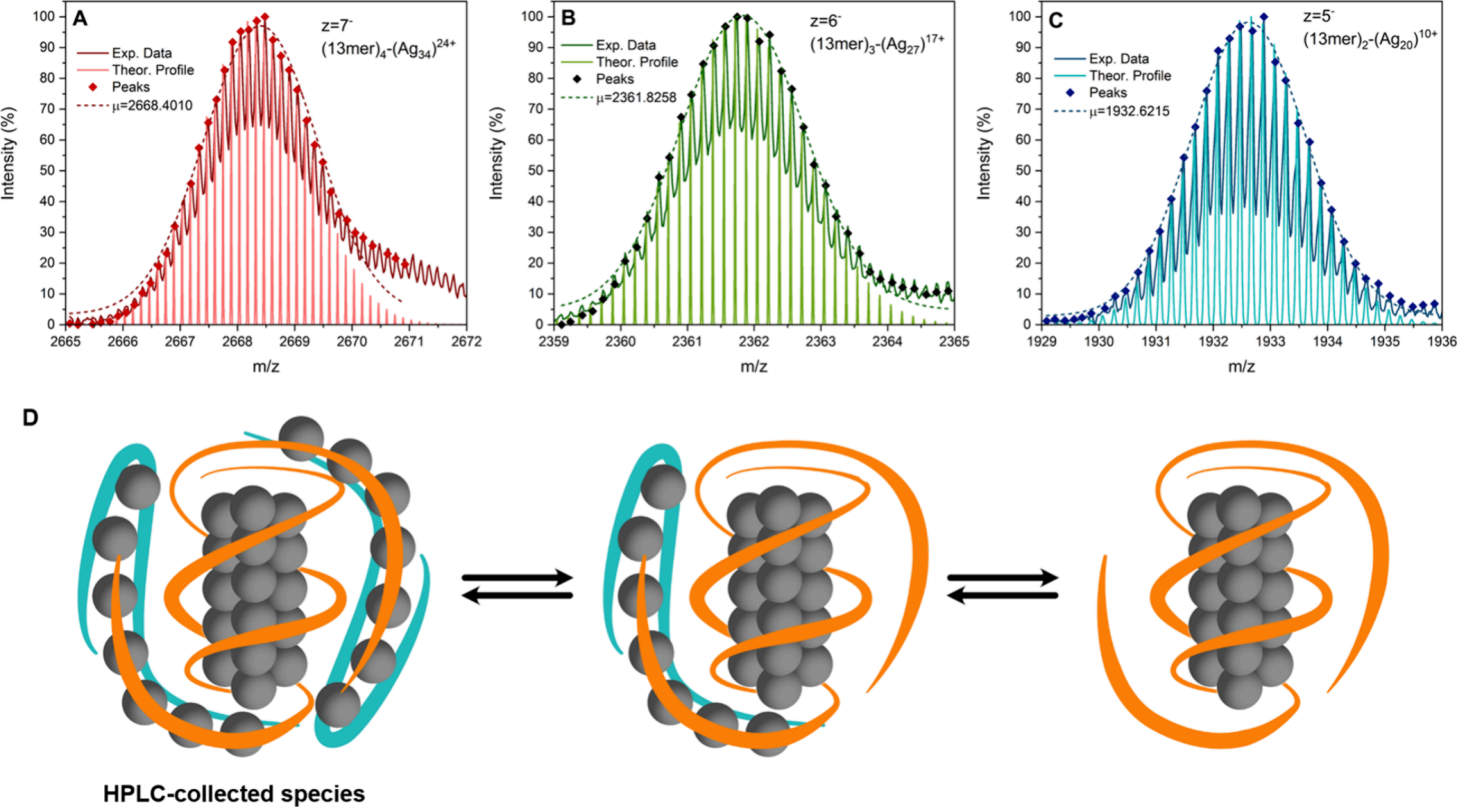
Mass spectrometry peaks of 13mer-AgNC related to (A) (13mer)_4_-[Ag_34_]^24+^ with *z* = 7^–^, (B) (13mer)_3_-[Ag_27_]^17+^ with *z* = 6^–^, and (C) (13mer)_2_-[Ag_20_]^10+^ with *z* = 5^–^. The experimental isotopic distribution is reported with the corresponding Gaussian fit and the theoretical isotopic distribution. The full mass spectrum and further peaks can be found in Figures S9 and S10. (A) The calculated average mass is *m*/*z* 2668.4010. The sum formula is C_480_H_608_N_168_O_300_P_48_Ag_34_, which corresponds to a molecular mass of 14176.6866 g/mol. (B) The calculated average mass is *m*/*z* 2361.8258. The sum formula is C_360_H_457_N_126_O_225_P_36_Ag_27_, which corresponds to a molecular mass of 14176.6866 g/mol. (C) The calculated average mass is *m*/*z* 1932.6215. The sum formula is C_240_H_306_N_84_O_150_P_24_Ag_20_, which corresponds to a molecular mass of 9668.2047 g/mol. (D) Cartoon-like model of the dynamic equilibria among (13mer)_4_-[Ag_34_]^24+^, (13mer)_3_-[Ag_27_]^17+^ and (13mer)_2_-[Ag_20_]^10+^.

The HPLC-collected fraction contains most likely the (13mer)_4_-[Ag_34_]^24+^ species, since samples are always up-concentrated before injection into the HPLC system (Figure 4D). In our previous work with isotopically pure DNA_2_-[Ag_16_Cl_2_]^8+^ nanoclusters, we have demonstrated dynamic atom exchange between AgNCs.^17^ We hypothesize that dynamic equilibria are also at play here between the (13mer)_2_-[Ag_20_]^10+^ and additional 13mer-[Ag_7_]^7+^ complexes, and the latter can reversibly dissociate by increasing the temperature or dilution, explaining the presence of (13mer)_2_-[Ag_20_]^10+^, (13mer)_3_-[Ag_27_]^17+^, and (13mer)_4_-[Ag_34_]^24+^ in the mass spectrometry data. It is worth noting that with 52 nucleotides and 34 Ag atoms, (13mer)_4_-[Ag_34_]^24+^ is the biggest isolated DNA-AgNC adduct to date. (13mer)_2_-[Ag_20_]^10+^ has 20 Ag atoms and 26 nucleobases; hence, if we assume that each nucleobase binds one Ag atom, six nucleobases are available to bind one or two 13mer-[Ag_7_]^7+^ complexes.^18,19^

The assumption that we collected mainly (13mer)_4_-[Ag_34_]^24+^ during HPLC is supported by measuring both the silver and phosphorus content by inductively coupled plasma-optical emission spectrometry (ICP-OES). The measured P:Ag ratio was found to be 1.4, consistent with the ratio expected from the (13mer)_4_-[Ag_34_]^24+^ species, since there are 12 P atoms per 13mer strand (Figures S11 and S12). Based on this ratio, we calculated a molar absorption coefficient of 169 000 M^–1^ cm^–1^ for (13mer)_4_-[Ag_34_]^24+^, which is similar to the value of 176 000 M^–1^ cm^–1^ found for the spectroscopically similar (16mer)_2_-[Ag_20_]^10+^.^7^ This finding further confirms that the two extra strands and the 14 Ag^+^ do not merge with the [Ag_20_]^10+^ core, since this would dramatically change the optical properties of the core compound.

Like the 13mer-AgNC, the emission spectra of 16mer-AgNC undergo similar, although less pronounced, changes with temperature (see Figure 1, as well as Figure S8), while varying the concentration does not influence either the absorption or the emission maxima of the 16mer-AgNC (Figure 2A). However, the concentration, and more specifically, the concentration of the stock solution before dilution, highly affects the limiting anisotropy value and the rotational correlation times. The fluorescence properties are typically measured by diluting a stock solution (100 μM ≤ [DNA-AgNC] ≤ 1 mM) of the DNA-AgNCs in order to keep the absorbance below 0.1 to avoid any possible inner filter effects. The more concentrated the stock solution is, the longer the rotational correlation time at 25 °C becomes. At the same time, the fundamental anisotropy (*r*_0_) value extracted at *t* = 0 deviates further (Figure S13) from the steady-state value of 0.39 measured in 99% glycerol at 5 °C (Figure S3A). In the case of very concentrated stock solutions, the anisotropy does not decay to zero within the fluorescence lifetime (Figure S13). This suggests that (16mer)_2_-[Ag_20_]^10+^ can form bulky aggregates, preventing the time-resolved anisotropy from decaying to zero within the measurement time.^20^ Additionally, it is worth mentioning that these aggregates take a significant amount of time to dissociate upon dilution, as rotational correlation times become shorter the more time elapses after dilution (data not shown).

On the other hand, in the case where the limiting anisotropy approaches 0.4 (Figure S14), the changes in the hydrodynamic volumes of the 16mer-AgNC indicate no significant amount of aggregates in the diluted sample. Similarly to the 13mer-AgNC, we attribute the measured changes (Figure 3B) to the binding of one or two 16mer strands with a range of Ag^+^ cations to the (16mer)_2_-[Ag_20_]^10+^ unit, which are also consistent with the mass spectrometry data presented below. It is also worth noting that the time-resolved anisotropy data at any temperature and concentration were fitted with one rotational correlation time, and hence reflect an averaged size. The idea of aggregates of 16mer-AgNCs is further supported by a recent study published by Chen et al., where an extended version of our 16mer sequence displayed the ability to dimerize.^10^

A previously published mass spectrum^7^ of 16mer-AgNC showed three *m*/*z* peaks between 2200 and 2400 that could be assigned to 2 DNA strands and [Ag_20_]^10+^, [Ag_21_]^11+^, and [Ag_22_]^12+^. Given the similarities in photophysical properties, it is reasonable to suggest that the core compound is [Ag_20_]^10+^ with either zero, one or two cationic silvers attached to nucleobases that do not interact with the [Ag_20_]^10+^ core. Furthermore, the value of 10 neutral Ag atoms agrees well with the observed NIR emission,^5^ and it is consistent with the results of Petty et al.^8^ and the value of 9.8 ± 0.2 reported by Copp and co-workers.^9^ It is indeed not unusual for DNA-AgNCs to have a slightly different number of Ag^+^ cations, as they do not usually determine the overall spectroscopic properties. By adjusting the mass spectrometry settings (see the Supporting Information for details), we observed not only peaks corresponding to 2 DNA strands with [Ag_20_]^10+^, [Ag_21_]^11+^ and [Ag_22_]^12+^, in agreement with the previously reported mass spectrum,^7^ but also three peaks related to an even larger number of Ag^+^ cations bound to (16mer)_2_-[Ag_20_]^10+^ (Figure 5A). These additional peaks are in line with the findings by Chen et al., where a significant number of additional Ag^+^ cations were observed to bind this repetitive C-rich sequence.^10^

**Figure 5:**
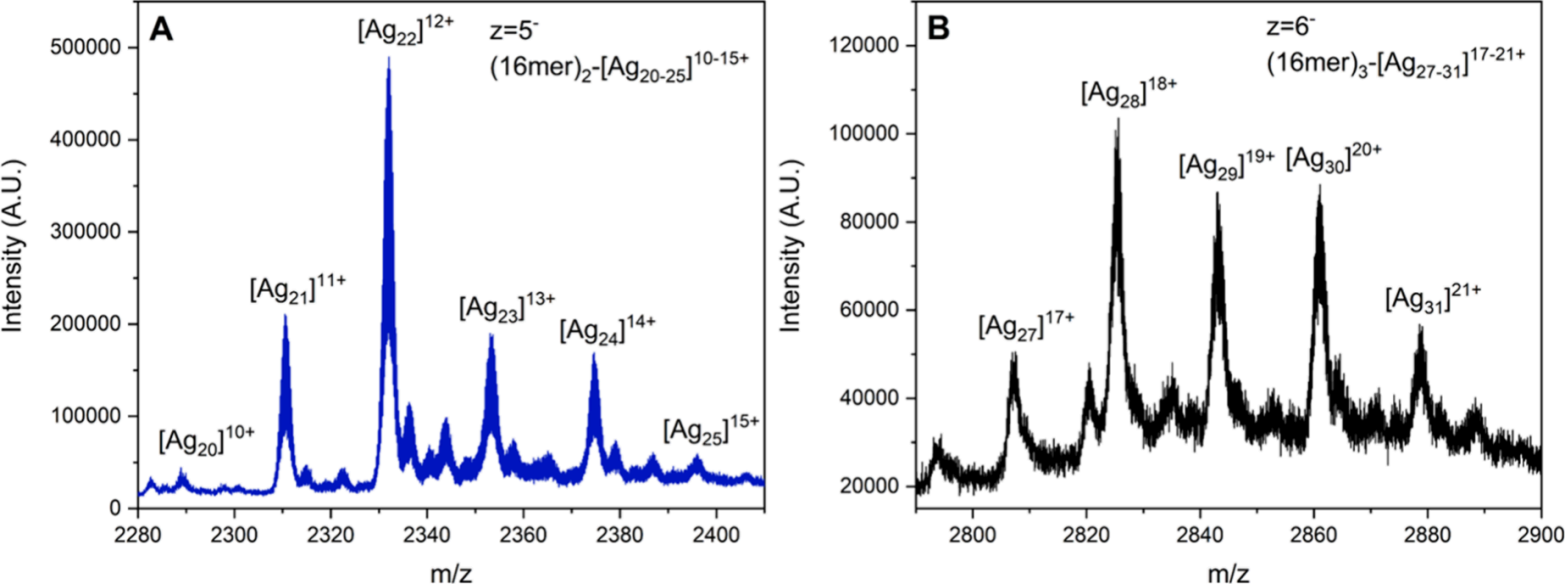
Mass spectrometry peaks of 16mer-AgNC related to (A) (16mer)_2_-[Ag_20–25_]^10–15+^ with *z* = 5^–^, and (B) (16mer)_3_-[Ag_27–31_]^17–21+^ with *z* = 6^–^. The full mass spectrum and the analysis of the peaks are reported in Figures S15, S16, S17, and S18.

Similarly to (13mer)_3_-[Ag_27_]^17+^, (16mer)_3_-[Ag_27–31_]^17–21+^ species were detected, supporting the idea of a (16mer)_2_-[Ag_20_]^10+^ unit able to complex another DNA strand via Ag^+^-mediated interactions (Figure 5B). This is consistent with the changes in the hydrodynamic volume shown in Figure 4B. Indeed, the extra nucleobases of the 16mer result in six additional free nucleobases compared to the 13mer case, bringing the total number of unbound nucleotides to 12. These DNA bases likely contributes to the large distribution of Ag^+^ adducts for both the (16mer)_2_-[Ag_20_]^10+^ and (16mer)_3_-[Ag_27_]^17+^ species observed in the mass spectrum. No mass spectrometry peaks corresponding to AgNC constructs with 4 DNA strands could be observed in this case, as these peaks are expected to appear above *m*/*z* 3700 for *z* = 6^–^ and 4400 for the 5^–^ charge state, which are beyond the detection limit of our mass spectrometer. Detailed analysis of the mass spectrum is reported in the Supporting Information. Moreover, the increased number of free nucleobases, combined with the cytosine-rich sequence, may not only promote the formation of (16mer)_3_-[Ag_27–31_]^17–21+^ species but also the formation of larger 16mer-AgNC aggregates in highly concentrated stock solutions (see the time-resolved anisotropy section above).

In summary, ESI-MS data of the 13mer-AgNC enabled the identification of three different (13mer)_2_-[Ag_20_]^10+^-based species corresponding to (13mer)_2_-[Ag_20_]^10+^, (13mer)_3_-[Ag_27_]^17+^, and (13mer)_4_-[Ag_34_]^24+^. Additionally, spectroscopic and time-resolved anisotropy data indicate that the 13mer-[Ag_7_]^7+^ segments can reversibly bind to (13mer)_2_-[Ag_20_]^10+^ depending on the temperature and concentration. For the 16mer-AgNC, ESI-MS data identified the presence of two main species, (16mer)_2_-[Ag_20_]^10+^ and (16mer)_3_-[Ag_27_]^17+^, each with a series of up to five Ag^+^ cations. The 16mer-AgNC sample shows less pronounced changes in the emissive properties with temperature, while concentration-dependent spectral shifts were not observed. Only the hydrodynamic volume and limiting anisotropy were affected by the concentration of the stock solution, which led us to hypothesize the additional formation of aggregates, due to the higher number of free nucleobases in the (16mer)_2_-[Ag_20_]^10+^ core unit, with respect to the 13mer-AgNC case. The unusual behavior of the two presented NIR emissive DNA-AgNCs could be related to the abundance of consecutive cytosines and the presence of several nucleobases not bound to the [Ag_20_]^10+^ core. These free DNA bases can indeed interact with other DNA strands via hydrogen bonding, π–π stacking, but most likely silver-mediated base pairing. Further investigations into the underlying mechanisms of this phenomenon could help fine-tune the photophysics of these fascinating emitters and provide valuable insights into nanomaterial design, especially in building larger assemblies of DNA-AgNCs.

## Supplementary Material



## Notes

HPLC chromatograms, absorption and emission spectra, time-correlated single photon counting data, anisotropy measurements, ICP-OES data, mass spectrometry data (PDF)
