# Supplementary material for: Dynamic Equilibria between DNA-Stabilized Silver Nanoclusters and Silver-Carrying DNA Strands
Source: J Phys Chem Lett. 2025 Oct 9;16(42):10856–61. doi: 10.1021/acs.jpclett.5c02324 (PMC12557354; doi:10.1021/acs.jpclett.5c02324)
Supplement: Supplementary file 1 [file jz5c02324_si_002.pdf]

Supporting Information

Dynamic Equilibria between DNA-stabilized Silver Nanoclusters and Silver-Carrying DNA Strands.

Cecilia Cerretani,[a,†,] Donato Ranieri,[b,†] Giacomo Romolini,[a] Christian Brinch Mollerup,[c] Letizia Liccardo,[a] Elena-Alexandra Niţă,[a] Loredana Latterini, [b] Tom Vosch [a,]**

[a] Department of Chemistry, University of Copenhagen, Universitetsparken 5, 2100 Copenhagen, Denmark.

[b] Department of Chemistry, Biology and Biotechnology, University of Perugia, Via Elce di Sotto, 8, 06123 Perugia, Italy.

[c] Department of Forensic Medicine, University of Copenhagen, Frederik V's Vej 11, 2100 Copenhagen, Denmark.

[†] These authors contributed equally.

Corresponding Author

* Cecilia Cerretani: cece@chem.ku.dk, Tom Vosch: tom@chem.ku.dk.

1. DNA-AgNCs Synthesis

The oligonucleotides (13mer: 5′–CCCACCCACCCTC–3′ and 16mer: 5′–CCCACCCACCCTCCCA–3′) were purchased from Integrated DNA Technologies (IDT). Silver nitrate ($AgNO_3$, ≥99.998%) and sodium borohydride ($NaBH_4$, 99.99%) were purchased from Sigma Aldrich. All chemicals were used as received. All solutions were prepared in nuclease-free water (IDT).

Both DNA-AgNCs, 13mer-AgNC and 16mer-AgNC, were synthesized by mixing the hydrated DNA with $AgNO_3$ in a 50 mM ammonium acetate (NH_4OAc) aqueous solution at pH 7. After 15 minutes, $NaBH_4$ was added in order to reduce the silver cations. The final ratio of the components was [DNA]:[Ag^+]:[BH_4^-] = 30 µM: 240 µM: 120 µM.

After storing the samples at 4 °C for three days, HPLC purification was performed, and the collected fractions were finally solvent-exchanged to 50 mM NH_4OAc aqueous solution by spin-filtration (cut-off membrane = 3 kDa).

2. HPLC Purification

The HPLC purification was performed using a preparative HPLC system from Agilent Technologies with an Agilent Technologies 1260 Infinity fluorescence detector and Agilent Technologies 1100 Series UV-Vis detector, and a Kinetex C18 column (5 µm, 100 Å, 250 × 4.6 mm, Phenomenex), equipped with a fraction collector. The mobile phase was a gradient mixture of 35 mM triethylammonium acetate (TEAA) buffer in water (A) and methanol (B).

The gradient was varied from 15% to 95% B as follows: 0-2 min 15% B, 2-17 min linear increase of B until 30%, 17-20 min from 30% to 95% B. The run was followed by 5 min of washing with 95% B to remove any traces of the sample from the column. The flow rate was 1 mL/min. The fraction collection was based on the absorption signal at 740 nm.

The chromatograms are shown in Figures S1 and S2.

3. Spectroscopic Measurements

Steady-state and time-resolved measurements were performed in 50 mM NH_4OAc solutions at diverse temperatures: 5, 25, and 40 °C for 16mer-AgNC and 10, 25, and 40 °C for 13mer-AgNC.

3.1 Absorption measurements

Absorption spectra were measured with either a Cary 300 UV-Vis spectrophotometer from Agilent Technologies or a Lambda1050 instrument (Perkin Elmer) using a deuterium lamp for ultraviolet radiation and a tungsten-halogen lamp for visible and near-infrared (NIR) radiation. The measurements were carried out in a single-beam configuration with a 0/100% transmittance baseline correction. Every spectrum was subtracted by the absorption spectrum of the corresponding blank (50 mM NH_4OAc).

3.2 Steady-state emission and anisotropy measurements

Steady-state fluorescence measurements were performed using a FluoTime300 instrument from PicoQuant. Fluorescence spectra and steady-state emission anisotropy data were recorded with a vertically-polarized 726-nm picosecond-pulsed lasers (LDH-P-C-730, PicoQuant). All emission spectra have been corrected for the wavelength dependency of the detector. Parallel (I_{VV}, I_{HH}) and perpendicular (I_{VH}, I_{HV}) emission spectra were recorded in 100 % glycerol at 5 °C.

These data were used to calculate the instrumental G factor ($G=I_{HV}/I_{HH}$) and the limiting anisotropy (r_0):

$$r_0 = \frac{I_{VV} - G*I_{VH}}{I_{VV} + 2G*I_{VH}} \quad (1)$$

3.2.1 *Quantum yield measurements and calculations*

Quantum yield (ϕ) values for 13mer-AgNCs was determined at 25 °C in 50 mM NH_4OAc, using Alexa 750 in a phosphate buffered saline solution (PBS) (ϕ_{ref}=0.12)[1] as reference dye. Absorption and emission spectra

of the clusters and reference compound were carried out at different concentrations in order to calculate the corresponding ϕ at each concentration, using the following formula:[2]

$$\boldsymbol{\Phi_{NC} = \frac{F_{NC}}{f_{A,NC}} \cdot slope_{ref}^{-1} \cdot \frac{n_{NC}^2}{n_{ref}^2} \cdot \Phi_{ref}} \quad \textbf{(2)}$$

where $\boldsymbol{\phi}$ represents the quantum yield, **F** is the integrated emission spectrum (*i.e.* the area under the fluorescence peak), $\mathbf{f_A}$ defines the fraction of absorbed light at the excitation wavelength (λ_{exc} =726 nm), and **n** is the refractive index of the medium where the clusters or the reference dye are dissolved in during the measurements. $\mathbf{slope_{ref}^{-1}}$ is given by $\frac{\mathbf{f_{A,ref}}}{\mathbf{F_{ref}}}$. The subscripts **NC** and **ref** indicate the DNA-AgNC and the reference compound, respectively.

3.2.2 *Time-correlated single photon counting (TCSPC) measurements and data analysis*

Time-resolved *fluorescence* and *anisotropy* measurements were performed exciting with a vertically-polarized (V) 726-nm pulsed laser.

Fluorescence decay curves were measured at 790 nm for 13mer-AgNC and 820 nm for 16mer-AgNC. The integration time was varied between 15 and 60 s, whereas the laser repetition rate was selected to 20.0 MHz in order to reach at least 10,000 counts in the maximum.

The analysis of time-resolved data was performed with FluoFit v.4.6 software from PicoQuant. All decay curves were globally fitted with a multi-exponential reconvolution model including the instrument response function (IRF). The obtained amplitude (α_i) and decay time (τ_i) components were used to calculate the intensity-averaged decay time, $<\tau>$.[3]

Time-resolved *anisotropy* measurements were carried out by recording parallel (VV, HH) and perpendicular (VH, HV) fluorescence decays at 790 nm for 13mer-AgNC and 820 nm for 16mer-AgNC. In order to reach at least 10,000 counts in the maximum, the integration time was set to 60 s and the repetition rate was chosen to be 20.0 MHz. The decay curves were fitted with FluoFit v.4.6 from PicoQuant using, respectively, a multi-exponential and a mono-exponential reconvolution model for the decay time and the rotational correlation time (θ), including the IRF.

The hydrodynamic volume (V_{hydro}) of the clusters (assumed to be spherical) was calculated at each temperature with the Perrin equation:[3]

$$\boldsymbol{\theta = \frac{\eta V_{hydro}}{k_B T}} \quad \textbf{(3)}$$

Where η is the dynamic viscosity of the solvent, k_B is the Boltzmann constant and T is the absolute temperature.

3.2.3 *Burst mode*

The luminescence traces in Figure S4B were measured for 16mer-AgNCs in 50 mM NH_4OAc at λ_{em} = 840 nm, exciting at 726 nm. The repetition rate of the laser was set to 40 MHz, with an effective sync rate of 500 Hz. The laser burst lasted 500 µs and consisted of 20000 pulses (*i.e.*, every pulse was separated by 25 ns) followed by 1.5 ms where the laser was switched off (25 % duty cycle). The ratio between the number of pulses and the repetition rate of the excitation laser defines the burst length, while the effective sync rate determines the overall length of the measurements (2 ms, in this case). The integration time was chosen to be 120 s.

4. Electrospray ionization-Mass Spectrometry (ESI-MS)

The ESI-MS data were acquired with a Xevo G2-XS QTof (Waters Corporation), using negative ion mode with a 1.5 kV capillary voltage, 30 V cone voltage, 80 V source offset and collision mode set to off. Spectra were collected from m/z 750 to 4000, with a scan time of 1 s. The source temperature was 100 °C with a

cone gas flow of 50 L/h, and the desolvation temperature and gas flow were 350 °C and 800 L/h, respectively. The QTOF was calibrated using ESI-L Low Tune Mix (Agilent Technologies), which contained compounds for negative mode in the mass range of m/z 113 to 2834. The sample was injected using an Acquity I-Class Plus system (Waters) with a flow-through needle autosampler, with a flow of 0.05 mL/min 50 mM NH_4OAc at pH 7 MeOH (80:20) and using 3 µL injection volume. The system was operated using UNIFI v.1.9.4 (Waters), and the final spectra were generated by averaging multiple spectra surrounding the apex of the observed peak. The recorded data were analyzed and fitted with the open-source software envipat (https://www.envipat.eawag.ch/).

Note on the selection of $(13mer)_4$-$[Ag_{34}]^{24+}$ *vs* $(13mer)_4$-$[Ag_{34}]^{23+}$. The table below shows the comparison between theoretical and experimental mass spectrometry peaks for $(13mer)_4$-$[Ag_{34}]^{X+}$ with X=23 and X=24. x_0 is the center of Gaussian fits for the experimentally measured mass spectra (x_0^{exp}) and the corresponding theoretical mass distributions (x_0^{th}). The last column corresponds to the absolute error calculated as x_0^{exp}-x_0^{th}.
While X=23 shows a smaller error compared to X=24, it is clear that the high noise level of the experimental data (Figures 4 and S10) and convolution with adducts' peaks affect the determination of the center of Gaussian fits for the experimentally measured mass spectra, x_0^{exp}. Indeed, when we set a threshold at 30% to remove the noise contribution, the error for X=24 is reduced. Furthermore, no solution-stable DNA-AgNCs with odd values of $Ag^{(0)}$ has been reported in the literature unless a slight miscalibration of the mass spectrometer was observed. The accurate assignment of the number of Ag^+ and $Ag^{(0)}$ relies on correctly determining the molecular weight of the compound down to a single proton; however this level of accuracy can be challenging to achieve by ESI-MS at high *m/z* values.[4] This is why we are confident in the assignment of a 24+ charge for the 13mer-AgNC species with 4 DNA strands and an overall number of silvers equals to 34.

**z**	**x_0^{exp}**		**Chemical formula**	**x_0^{th}**	**error**
**7^-** (Figure 4A)	**All peaks**	2668.4010	$C_{480}H_{632}N_{168}O_{300}P_{48}[Ag_{34}]^{24+}$	2668.2930	+ 0.1080
			$C_{480}H_{632}N_{168}O_{300}P_{48}[Ag_{34}]^{23+}$	2668.4015	- 0.0005
	**> 30%**	2668.3578	$C_{480}H_{632}N_{168}O_{300}P_{48}[Ag_{34}]^{24+}$	2668.2930	+ 0.0648
			$C_{480}H_{632}N_{168}O_{300}P_{48}[Ag_{34}]^{23+}$	2668.4015	- 0.0437
**6^-** (Figure S8B)	**All peaks**	3113.3299	$C_{480}H_{632}N_{168}O_{300}P_{48}[Ag_{34}]^{24+}$	3113.1734	+ 0.1565
			$C_{480}H_{632}N_{168}O_{300}P_{48}[Ag_{34}]^{23+}$	3113.3027	+ 0.0272
	**> 30%**	3113.2537	$C_{480}H_{632}N_{168}O_{300}P_{48}[Ag_{34}]^{24+}$	3113.1734	+ 0.0803
			$C_{480}H_{632}N_{168}O_{300}P_{48}[Ag_{34}]^{23+}$	3113.3027	- 0.0490

5. <u>Molar absorption coefficient (ε) determination</u>

The molar absorption coefficient of 13mer-AgNC was determined as reported by Romolini *et al.* employing inductively coupled plasma-optical emission spectrometry (ICP-OES).[5] The ICP-OES calibration curves for silver and phosphorus are reported in Figures S11 and S12. The ε value at 750 nm was found to be 169000 M^{-1} cm^{-1}.

References

[1] AAT Bioquest https://www.aatbio.com
[2] A. M. Brouwer; *Pure Appl. Chem.*, **2011**, *83*, 2213.
[3] J. R. Lakowicz; *Principles of Fluorescence Spectroscopy*, **2006**.
[4] R. Guha *et al.*, *Nanoscale*, **2024**, *16*, 20596-20607.
[5] G. Romolini, G. *et al.*, *Nanoscale,* **2024**, *16*, 12559-12566.

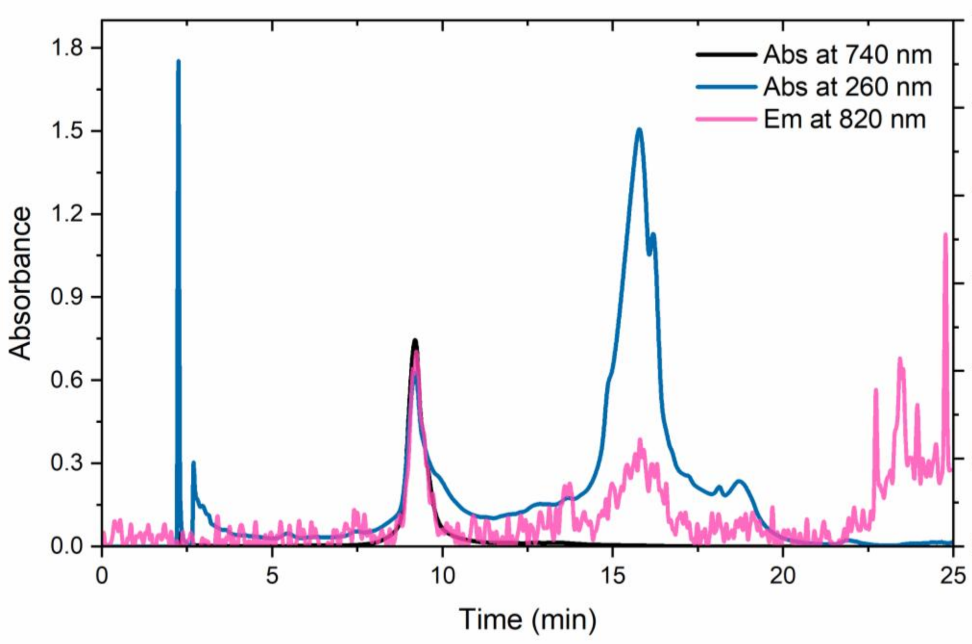

Figure S1. *HPLC chromatograms of 13mer-AgNCs monitoring the absorption at 740 nm (black) and 260 nm (blue), and the fluorescence signal at 820 nm, exciting at 740 nm (pink). The fraction collected at 9 min was used in the study.*

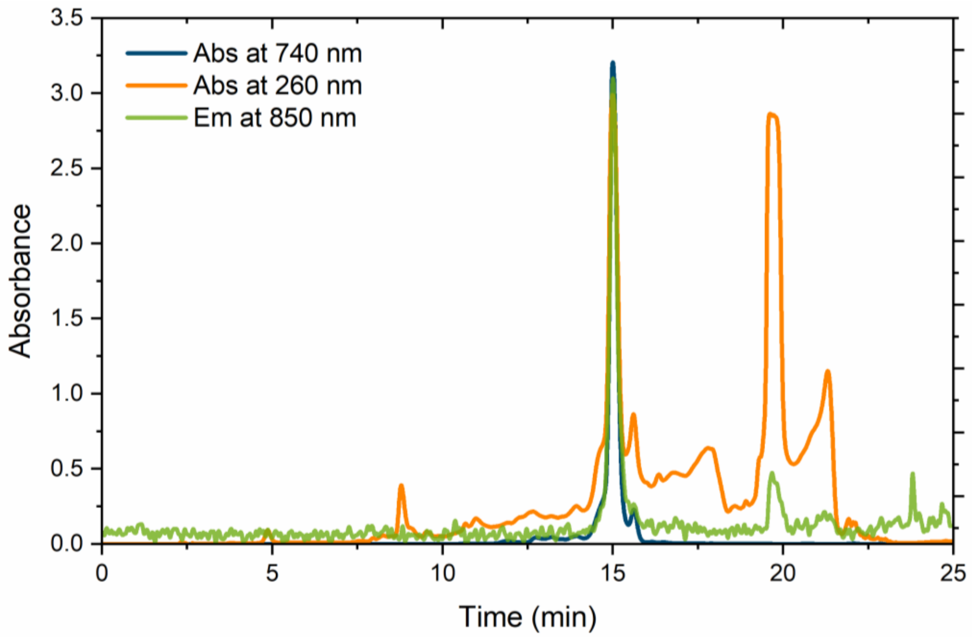

Figure S2. *HPLC chromatograms of 16mer-AgNCs monitoring the absorption at 740 nm (blue) and 260 nm (orange), and the fluorescence signal at 850 nm, exciting at 740 nm (green). The fraction collected at 15 min was used in the study.*

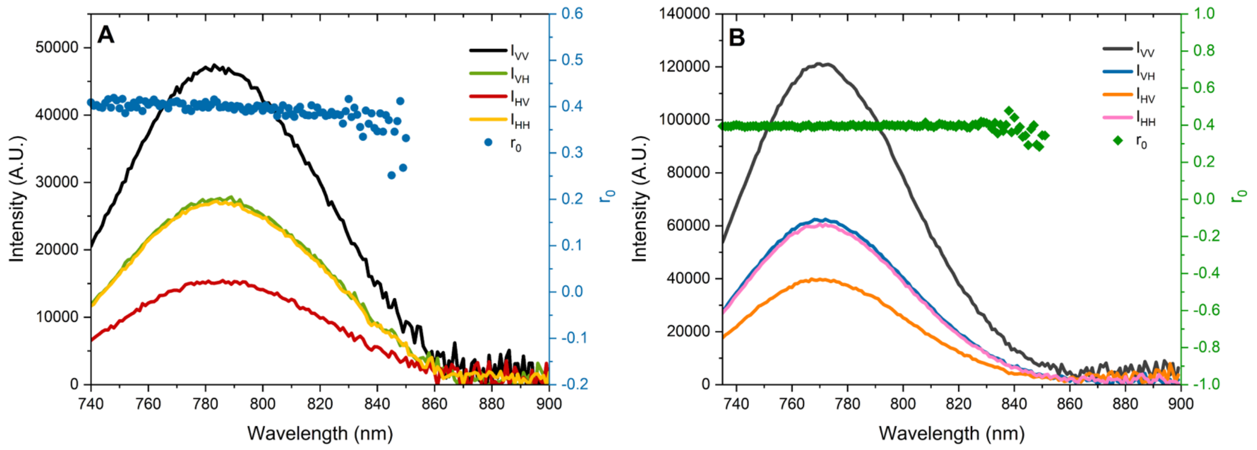

Figure S3. *Steady-state emission anisotropy data for* ***(A)*** *16mer-AgNCs and* ***(B)*** *13mer-AgNCs in 100% glycerol at 5 ˚C. Vertical-vertical (I_{VV}), vertical-horizontal (I_{VH}), horizontal-vertical (I_{HV}), and horizontal-horizontal (I_{VV}) emission spectra together with the limiting anisotropy traces (r_0).*

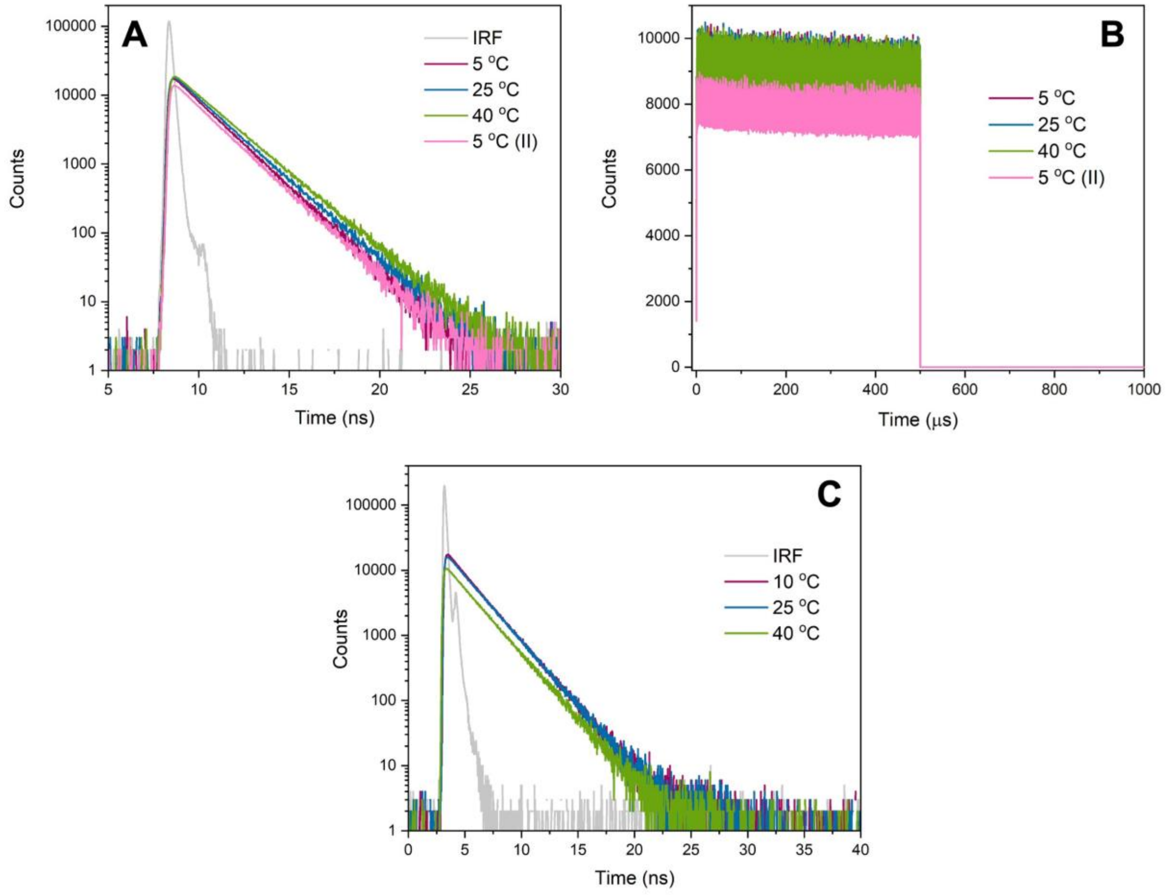

Figure S4. ***(A)*** *Fluorescence decays collected at 820 nm and* ***(B)*** *luminescence traces measured at 840 nm of 16mer-AgNCs in 50 mM NH_4OAc at 5, 25, 40, and 5 (II) ˚C. The average fluorescence decay times in (A) are 1.73, 1.82, 1.95, and 1.74 ns, respectively.* ***(C)*** *Fluorescence decays taken at 790 nm of 13mer-AgNCs in 50 mM NH4OAc at 10, 25, and 40 °C. All samples were excited at 726 nm.*

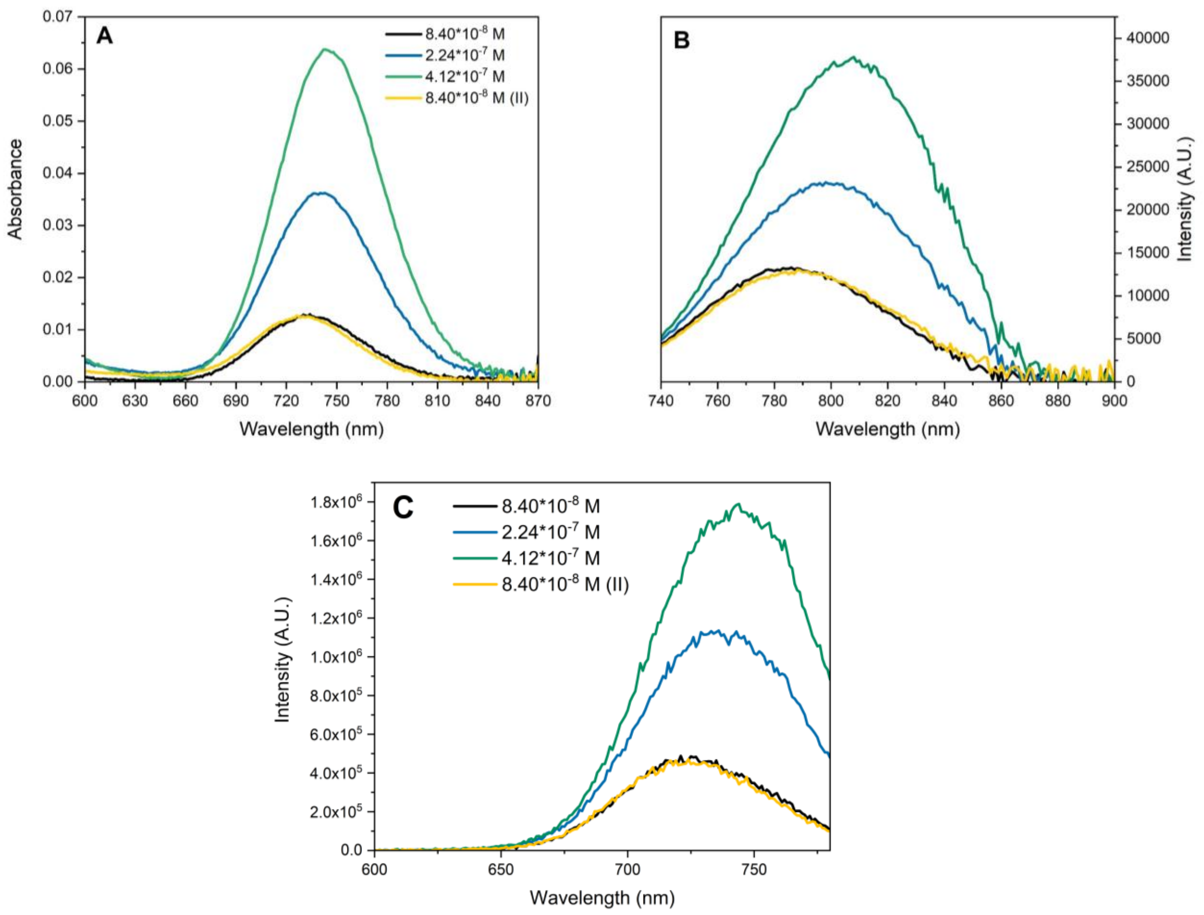

Figure S5. (A) *Absorption,* ***(B)*** *emission and* ***(C)*** *excitation spectra of 13mer-AgNCs in 50 mM NH_4OAc at 25 ˚C at different concentrations (λ_{exc}=726 nm and λ_{em}=790 nm, respectively). (II) indicates that the sample was diluted to match the initial concentration.*

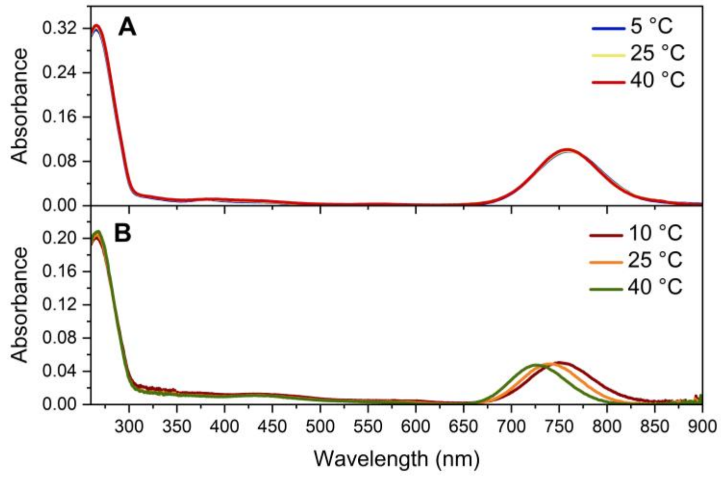

Figure S6. *Exteded version (260 – 900 nm) of the absorption spectra of* ***(A)*** *16mer-AgNCs and* ***(B)*** *13mer-AgNCs in 50 mM NH_4OAc, displayed in Figure 1*

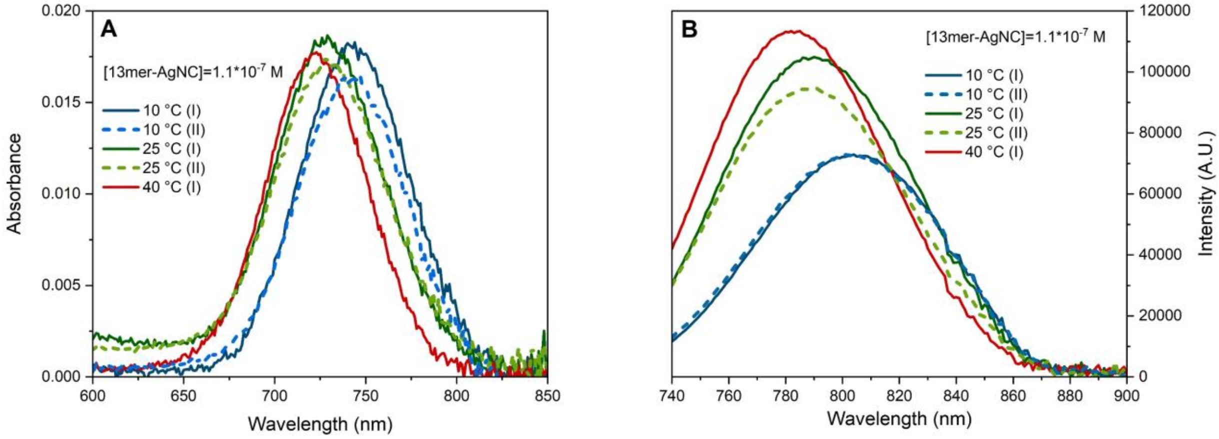

Figure S7. (A) *Absorbance and (**B**) emission spectra of 13mer-AgNCs in 50 mM NH_4OAc at different temperatures (λ_{exc}=726 nm). The concentration of the sample was set to $1.1{\cdot}10^{-7}$ M. (II) indicates the backward temperature cycle.*

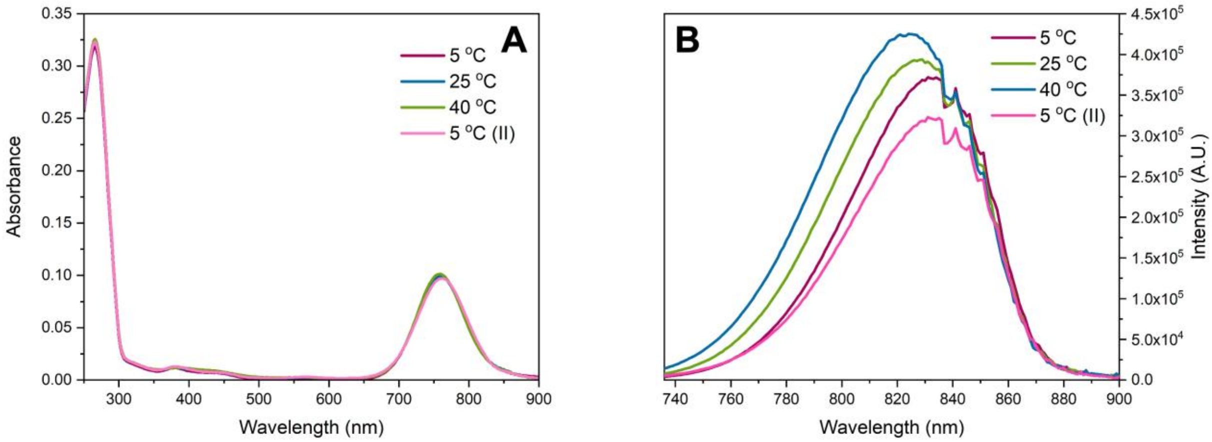

Figure S8. (A) *Absorbance and (**B**) emission spectra of 16mer-AgNCs in 50 mM NH_4OAc at different temperatures (λ_{exc}=726 nm). The concentration of 16mer-AgNCs is $5.6{\cdot}10^{-7}$ M.*

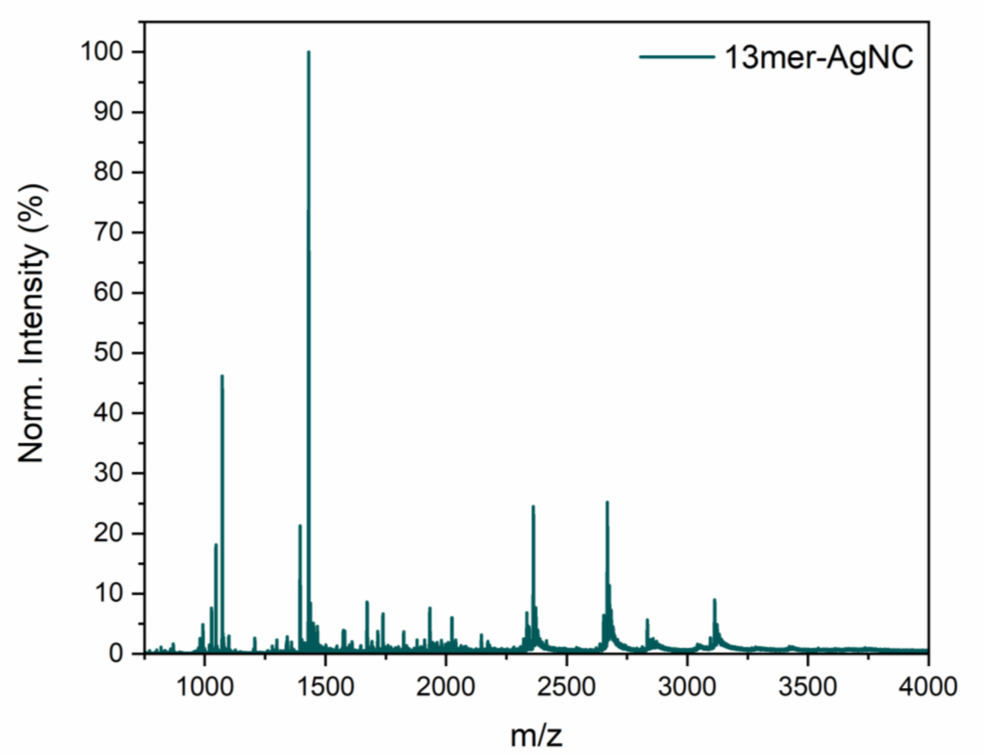

Figure S9. *Mass spectrum of 13mer-AgNCs measured in negative ion mode.*

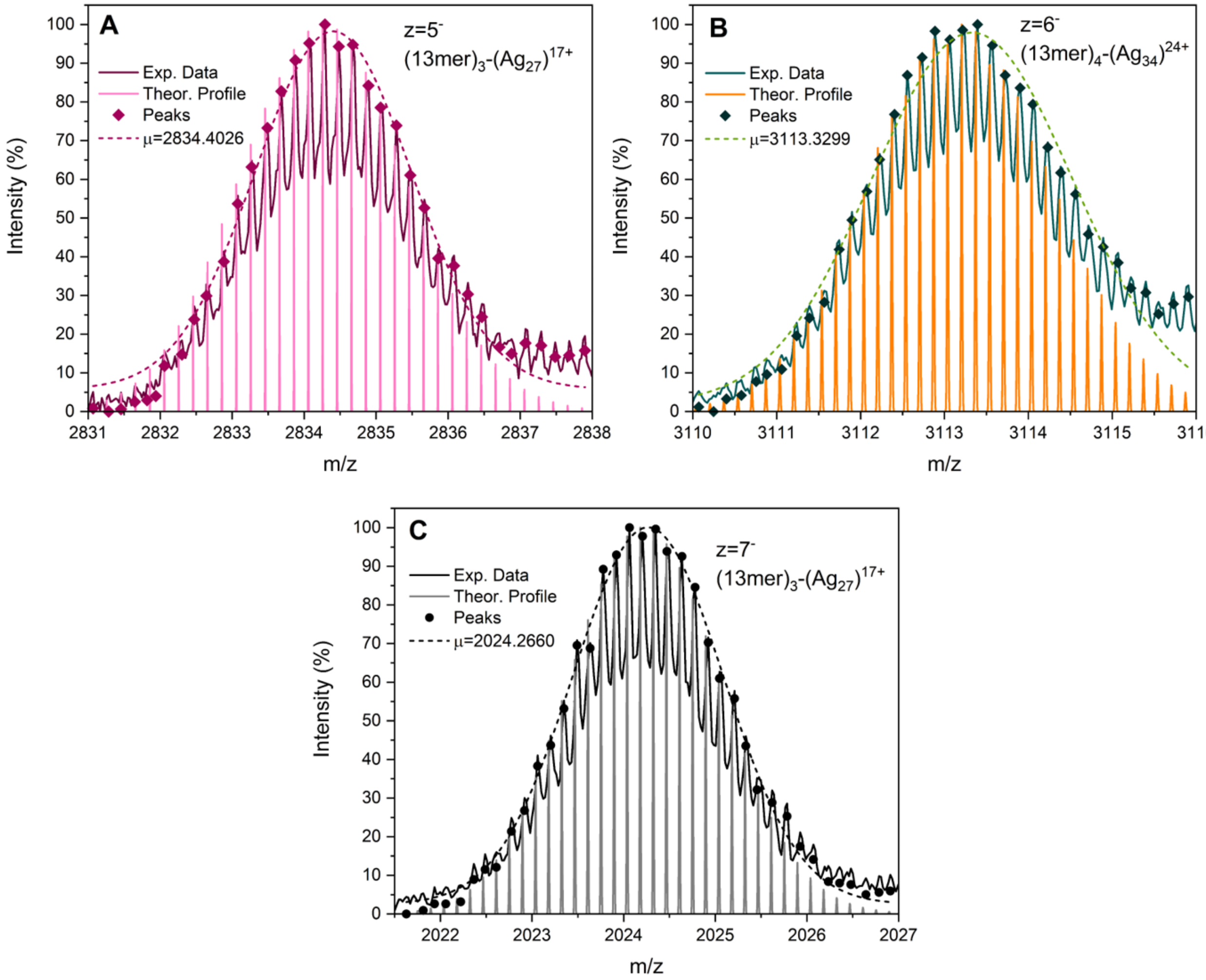

Figure S10. *Mass spectrometry peaks of 13mer-AgNC. The experimental isotopic distribution is reported with the corresponding Gaussian fit and the theoretical isotopic distribution. (A) (13mer)$_3$-[Ag$_{27}$]$^{17+}$ with z=5$^-$, (B) (13mer)$_4$-[Ag$_{34}$]$^{24+}$ with z=6$^-$, and (C) (13mer)$_3$-[Ag$_{27}$]$^{17+}$ with z=7$^-$. (A) The calculated average mass is m/z 2834.4026. The sum formula is $C_{360}H_{457}N_{126}O_{225}P_{36}Ag_{27}$, which corresponds to a molecular mass of 14176.6866 g/mol. (B) The calculated average mass is m/z 3113.3299. The sum formula is $C_{480}H_{608}N_{168}O_{300}P_{48}Ag_{34}$, which corresponds to a molecular mass of 18685.1684 g/mol. (C) The calculated average mass is m/z 2024.2660. The sum formula is $C_{360}H_{457}N_{126}O_{225}P_{36}Ag_{27}$, which corresponds to a molecular mass of 14176.6866 g/mol.*

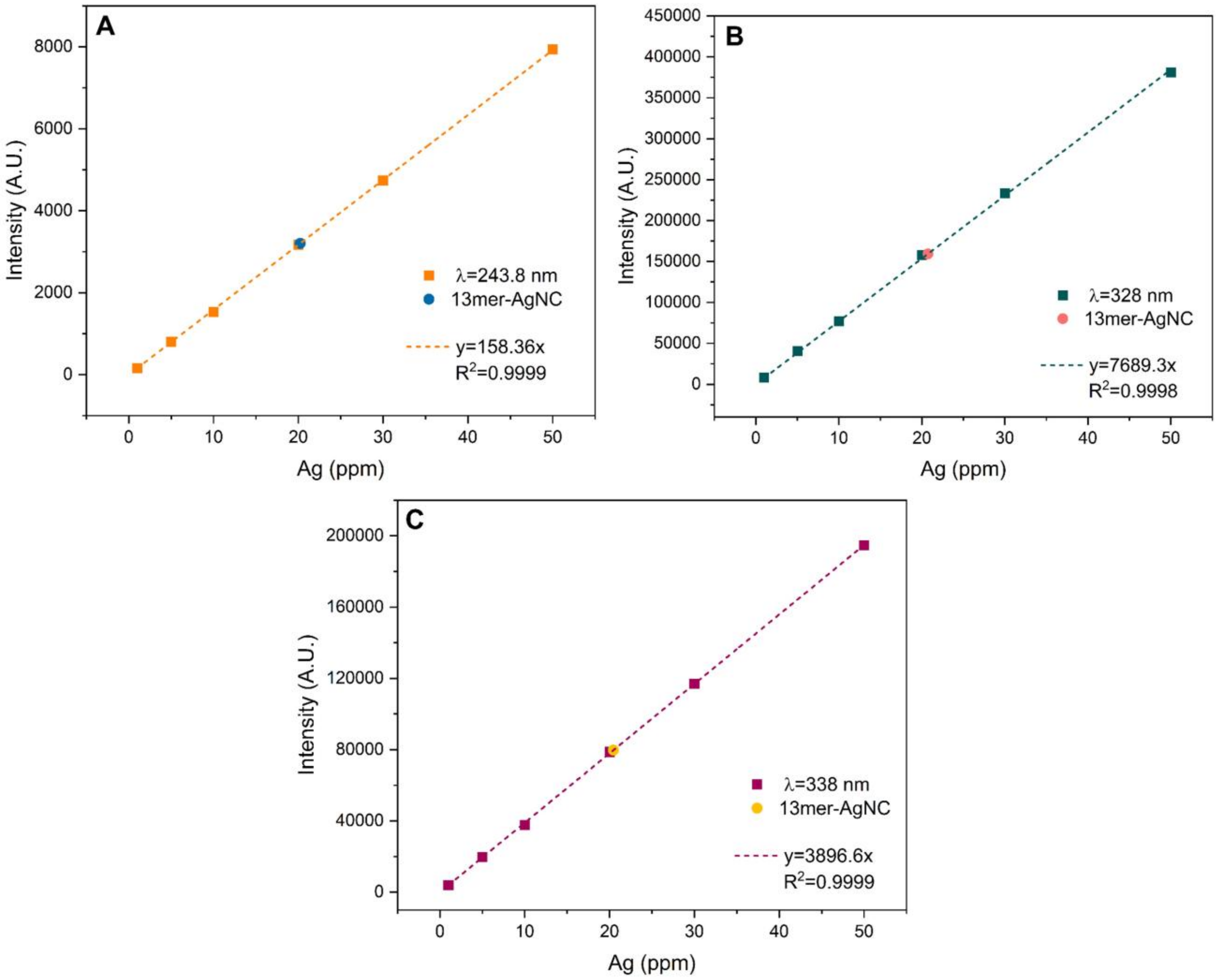

Figure S11. *ICP-OES calibration curves for 13mer-AgNCs using different emission lines of silver:* ***(A)*** *243.8 nm,* ***(B)*** *328 nm, and* ***(C)*** *338 nm. The circle indicates the amount of Ag (ppm) in the 13mer-AgNC sample. An average* ***Ag ppm*** *value of* ***20.47*** *was found, which corresponds to* ***[Ag]=189.73 µM****.*

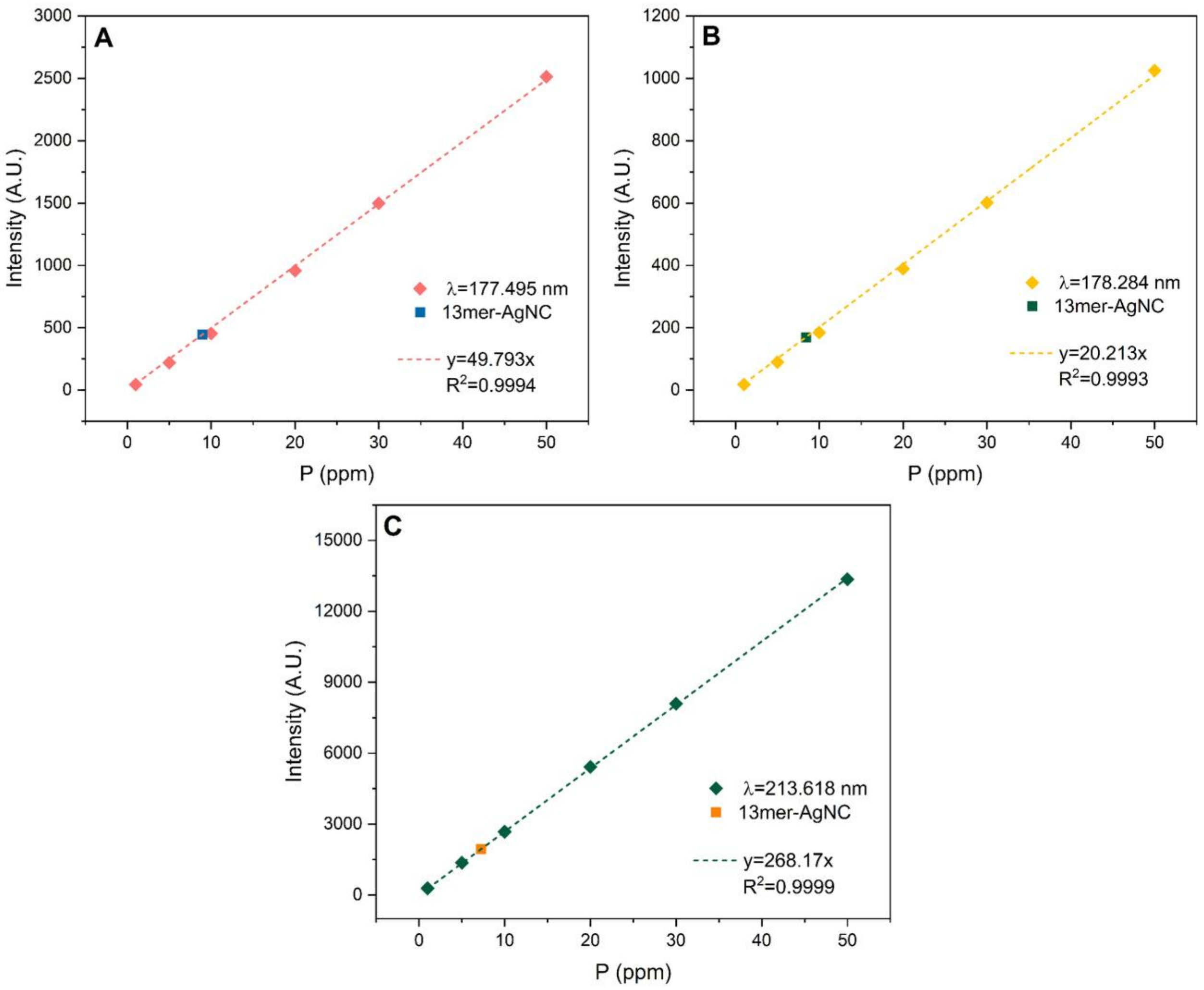

Figure S12. *ICP-OES calibration curves for 13mer-AgNCs using different emission lines of phosphorus:* ***(A)*** *177.495 nm,* ***(B)*** *178.284 nm, and* ***(C)*** *213.618 nm. The square indicates the amount of P (ppm) in the 13mer-AgNC sample. An average* ***P ppm*** *value of* ***8.203*** *was found, which corresponds to* ***[P]= 264.87 µM****.*

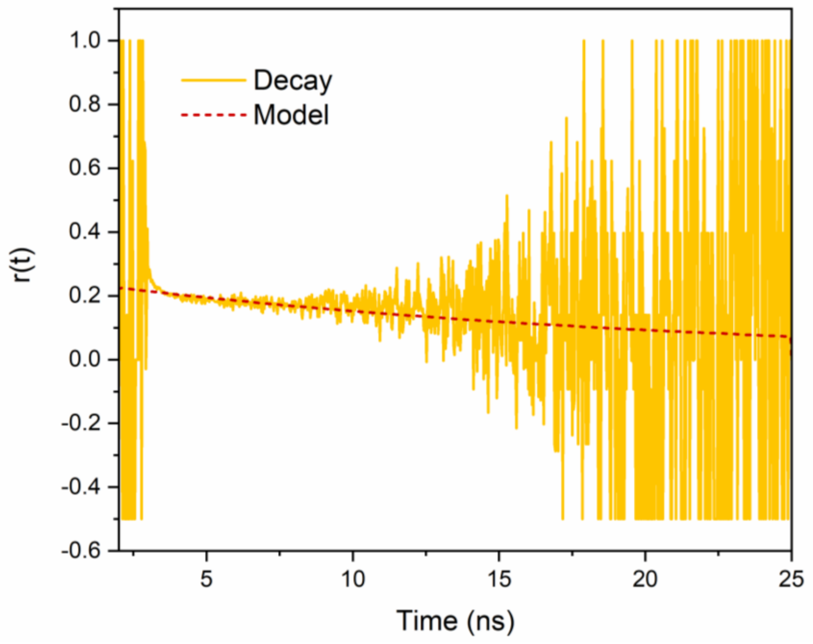

Figure S13. *Anisotropy decay of 16mer-AgNCs in 50 mM* NH_4OAc *at 25 ˚C in case of large amount of aggregates. The extrapolated* r_0 *value corresponds to 0.23 and the calculated hydrodynamic volume is 84.6* nm^3 *given a rotational correlation time of 18.27 ns. r(t) does not reach zero within the measurement window.*

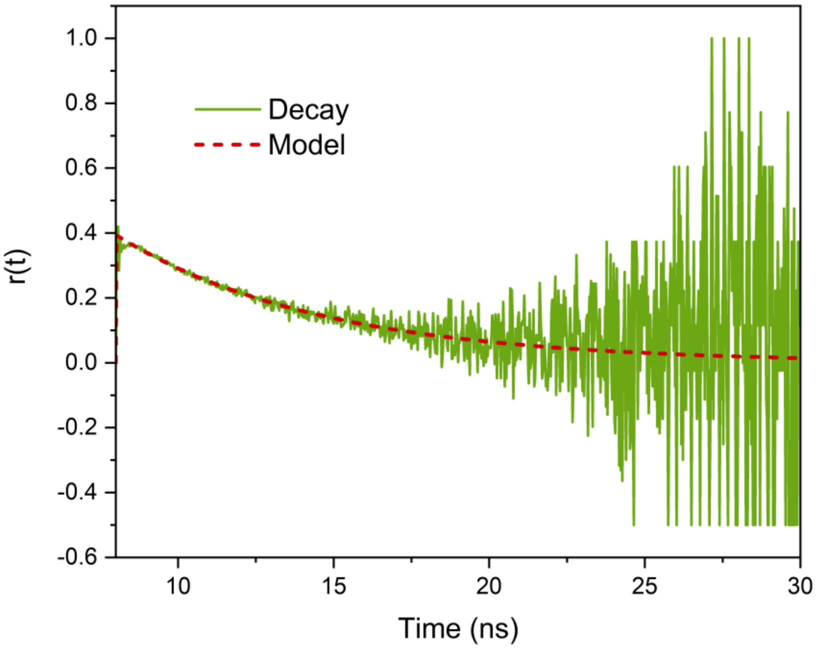

Figure S14. *Anisotropy decay of 16mer-AgNCs in 50 mM* NH_4OAc *at 25 ˚C in case of a limited amount of aggregates. The extrapolated* r_0 *value corresponds to 0.39 and the calculated hydrodynamic volume is 35.2* nm^3 *(Figure 4B) given a rotational correlation time of 7.612 ns. r(t) is approaching zero within the measurement window.*

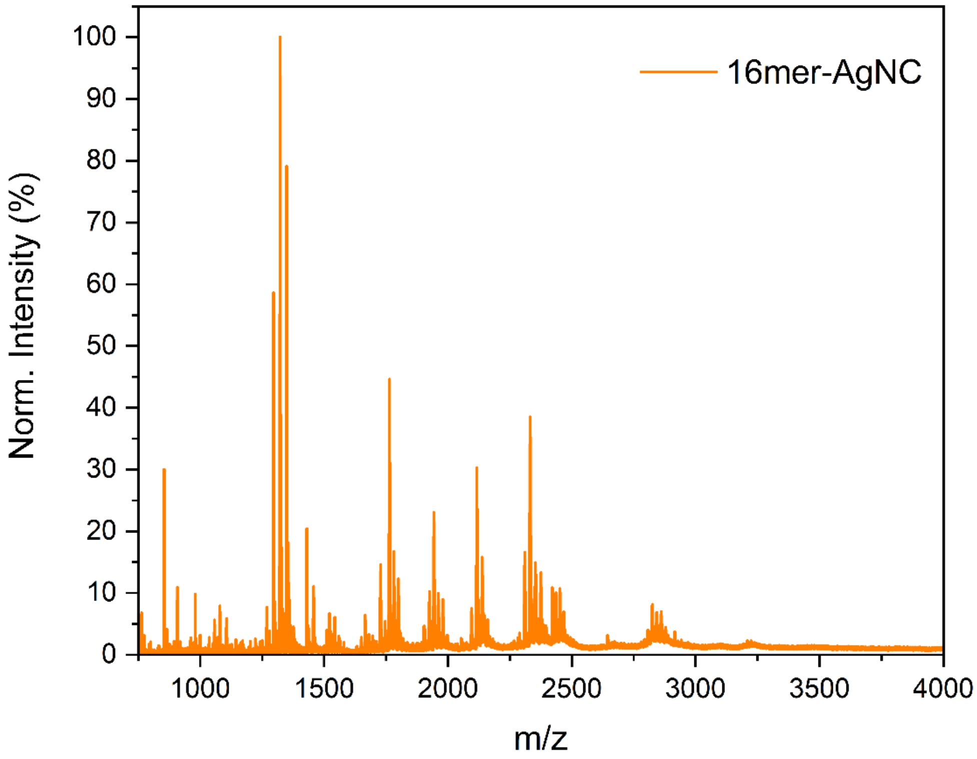

Figure S15. *Mass spectrum of 16mer-AgNCs measured in negative ion mode.*

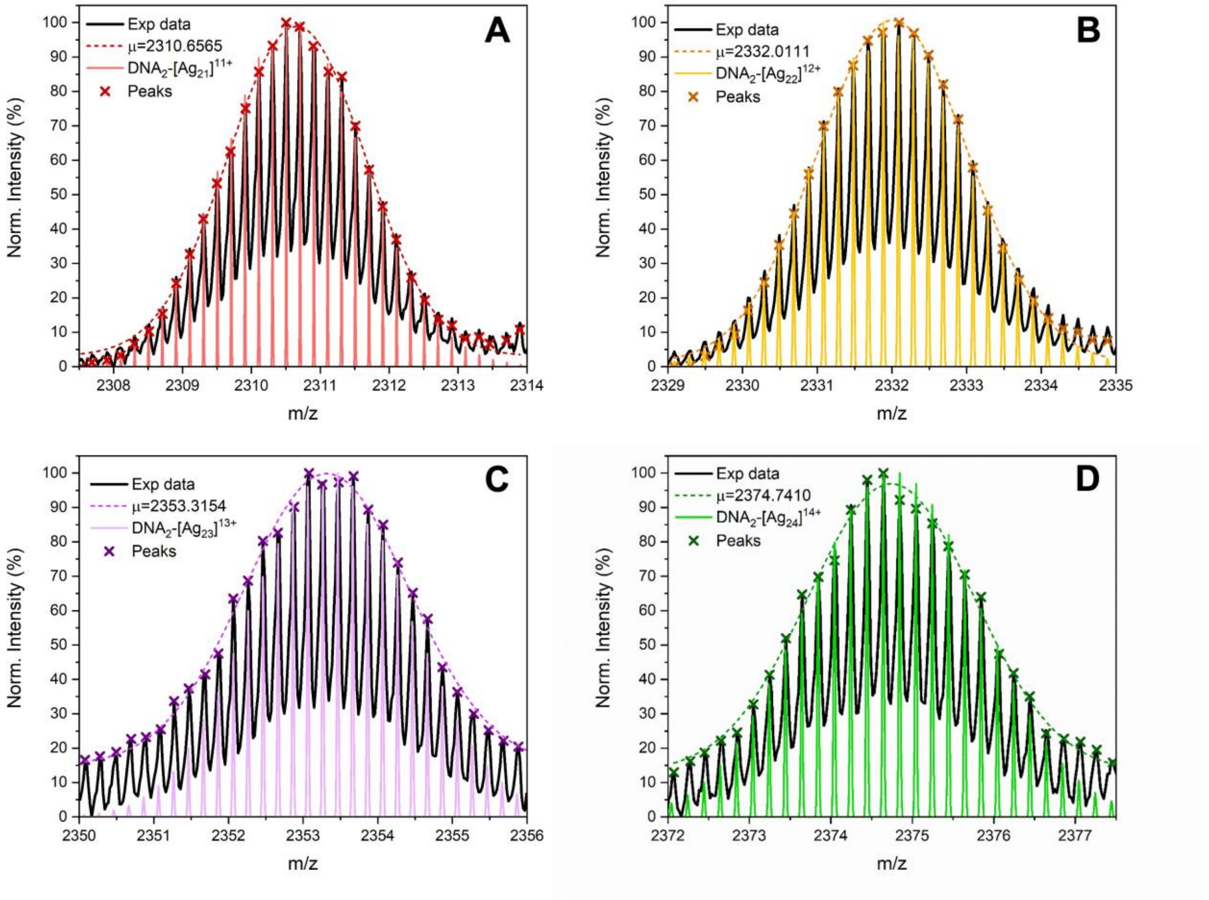

Figure S16. *Mass spectrometry peaks of 16mer-AgNC related to $z=5^-$. The experimental isotopic distribution is reported with the corresponding Gaussian fit and the theoretical isotopic distribution.* ***(A)*** *$(16mer)_2$-$[Ag_{21}]^{11+}$,* ***(B)*** *$(16mer)_2$-$[Ag_{22}]^{12+}$,* ***(C)*** *$(16mer)_2$-$[Ag_{23}]^{13+}$ and* ***(D)*** *$(16mer)_2$-$[Ag_{24}]^{14+}$.* ***(A)*** *The calculated average mass is m/z 2310.6565. The sum formula is $C_{296}H_{377}N_{106}O_{184}P_{30}Ag_{21}$, which corresponds to a molecular mass of 11558.2054 g/mol.* ***(B)*** *The calculated average mass is m/z 2332.0111. The sum formula is $C_{296}H_{376}N_{106}O_{184}P_{30}Ag_{22}$, which corresponds to a molecular mass of 11665.0657 g/mol.* ***(C)*** *The calculated average mass is m/z 2353.3154. The sum formula is $C_{296}H_{375}N_{106}O_{184}P_{30}Ag_{23}$, which corresponds to a molecular mass of 11771.9259 g/mol.* ***(D)*** *The calculated average mass is m/z 2374.7410. The sum formula is $C_{296}H_{374}N_{106}O_{184}P_{30}Ag_{24}$, which corresponds to a molecular mass of 11878.7862 g/mol.*

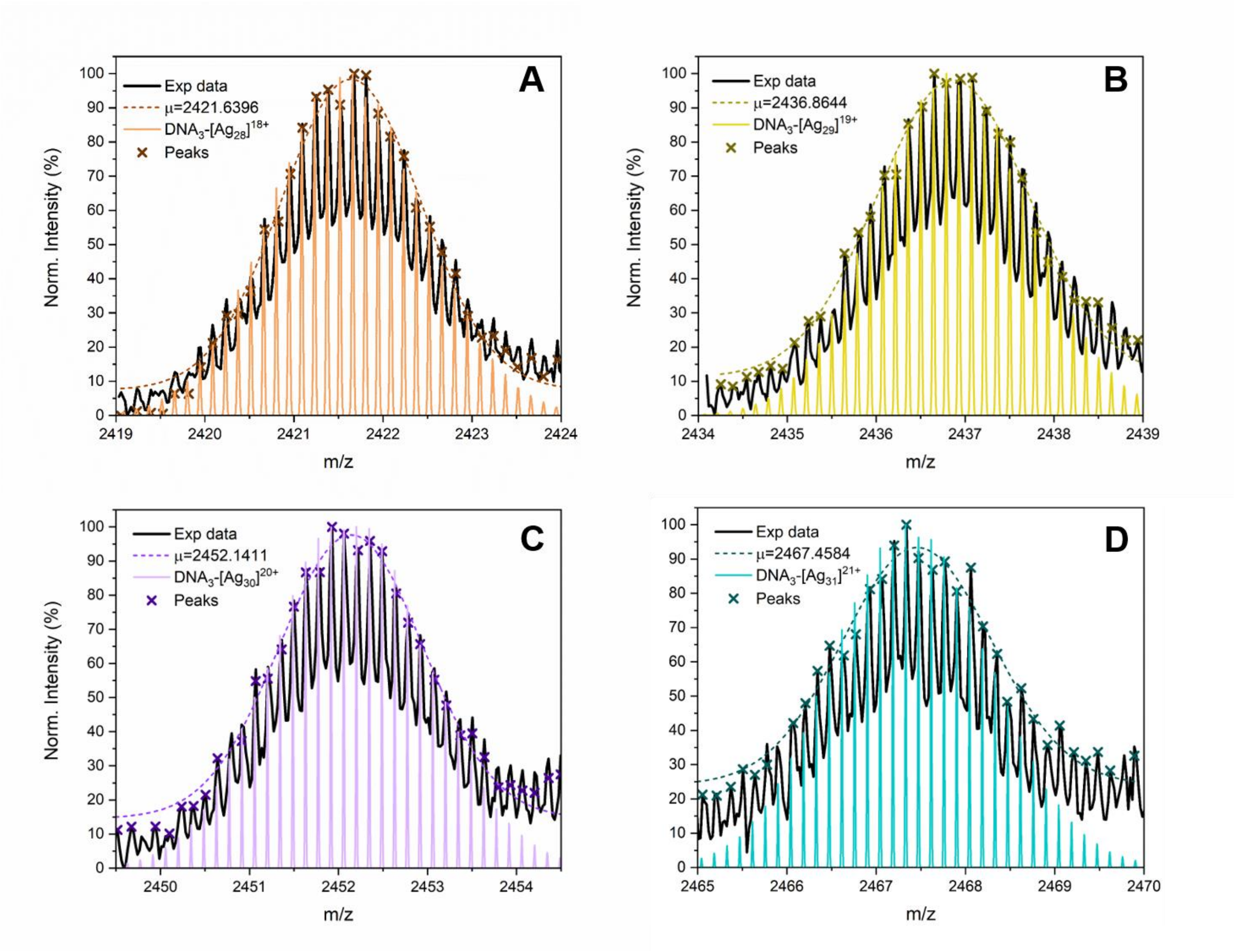

Figure S17. *Mass spectrometry peaks of 16mer-AgNC related to z=7*$^{-}$*. The experimental isotopic distribution is reported with the corresponding Gaussian fit and the theoretical isotopic distribution.* ***(A)*** *(16mer)*$_3$*-[Ag*$_{28}$*]*$^{18+}$*,* ***(B)*** *(16mer)*$_3$*-[Ag*$_{29}$*]*$^{19+}$*,* ***(C)*** *(16mer)*$_3$*-[Ag*$_{30}$*]*$^{20+}$ *and* ***(D)*** *(16mer)*$_3$*-[Ag*$_{31}$*]*$^{21+}$*.* ***(A)*** *The calculated average mass is m/z 2421.6396. The sum formula is* $C_{444}H_{564}N_{159}O_{276}P_{45}Ag_{28}$*, which corresponds to a molecular mass of 16958.2575 g/mol.* ***(B)*** *The calculated average mass is m/z 2436.8644. The sum formula is* $C_{444}H_{563}N_{159}O_{276}P_{45}Ag_{29}$*, which corresponds to a molecular mass of 17065.1178 g/mol.* ***(C)*** *The calculated average mass is m/z 2452.1411. The sum formula is* $C_{444}H_{562}N_{159}O_{276}P_{45}Ag_{30}$*, which corresponds to a molecular mass of 17171.9780 g/mol.* ***(D)*** *The calculated average mass is m/z 2467.4584. The sum formula is* $C_{444}H_{561}N_{159}O_{276}P_{45}Ag_{31}$*, which corresponds to a molecular mass of 17278.8383 g/mol.*

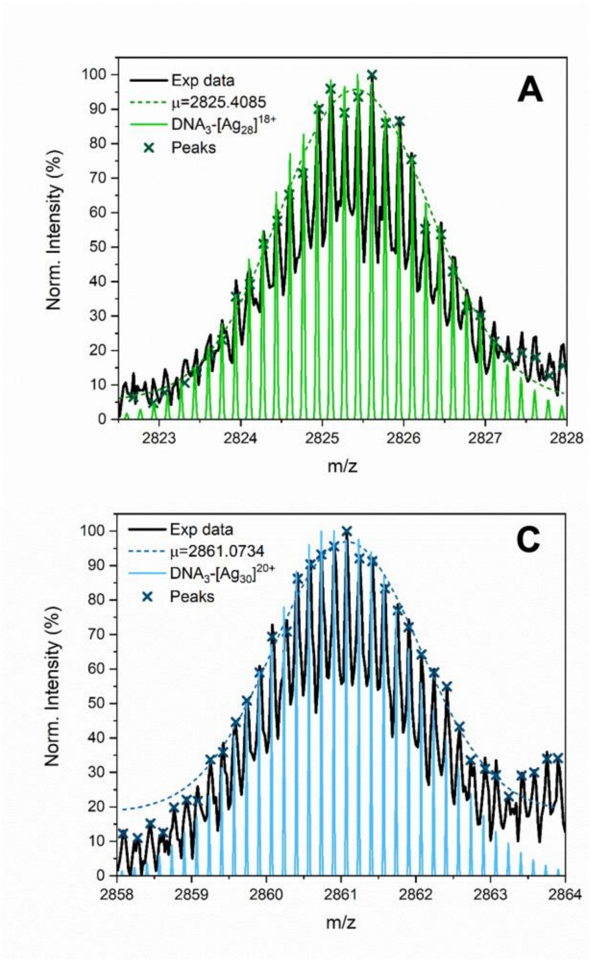

Figure S18. *Mass spectrometry peaks of 16mer-AgNC related to z=6*$^{-}$. *The experimental isotopic distribution is reported with the corresponding Gaussian fit and the theoretical isotopic distribution.* ***(A)*** *(16mer)*$_3$*-[Ag*$_{28}$*]*$^{18+}$*,* ***(B)*** *(16mer)*$_3$*-[Ag*$_{29}$*]*$^{19+}$*,* ***(C)*** *(16mer)*$_3$*-[Ag*$_{30}$*]*$^{20+}$ *and* ***(D)*** *(16mer)*$_3$*-[Ag*$_{31}$*]*$^{21+}$*.* ***(A)*** *The calculated average mass is m/z 2825.4085. The sum formula is* $C_{444}H_{564}N_{159}O_{276}P_{45}Ag_{28}$*, which corresponds to a molecular mass of 16958.2575 g/mol.* ***(B)*** *The calculated average mass is m/z 2843.2165. The sum formula is* $C_{444}H_{563}N_{159}O_{276}P_{45}Ag_{29}$*, which corresponds to a molecular mass of 17065.1178 g/mol.* ***(C)*** *The calculated average mass is m/z 2861.0734. The sum formula is* $C_{444}H_{562}N_{159}O_{276}P_{45}Ag_{30}$*, which corresponds to a molecular mass of 17171.9780 g/mol.* ***(D)*** *The calculated average mass is m/z 2878.7496. The sum formula is* $C_{444}H_{561}N_{159}O_{276}P_{45}Ag_{31}$*, which corresponds to a molecular mass of 17278.8383 g/mol.*
